# Reactive Oxygen Species Function to Mediate the Fe Deficiency Response in an Fe-Efficient Apple Genotype: An Early Response Mechanism for Enhancing Reactive Oxygen Production

**DOI:** 10.3389/fpls.2016.01726

**Published:** 2016-11-16

**Authors:** Chaohua Sun, Ting Wu, Longmei Zhai, Duyue Li, Xinzhong Zhang, Xuefeng Xu, Huiqin Ma, Yi Wang, Zhenhai Han

**Affiliations:** ^1^Institute for Horticultural Plants, College of Horticulture, China Agricultural UniversityBeijing, China; ^2^Key Laboratory of Physiology and Molecular Biology of Tree Fruit of Beijing, China Agricultural UniversityBeijing, China; ^3^Beijing Collaborative Innovation Center for Eco-environmental Improvement with Forestry and Fruit Trees, China Agricultural UniversityBeijing, China

**Keywords:** ROS, Fe deficiency, early response, *Malus xiaojinensis*, woody plant

## Abstract

Reactive oxygen species (ROS) are important signaling molecules in plants that contribute to stress acclimation. This study demonstrated that ROS play a critical role in Fe deficiency-induced signaling at an early stage in *Malus xiaojinensis*. Once ROS production has been initiated, prolonged Fe starvation leads to activation of ROS scavenging mechanisms. Further, we demonstrated that ROS scavengers are involved in maintaining the cellular redox homeostasis during prolonged Fe deficiency treatment. Taken together, our results describe a feedback repression loop for ROS to preserve redox homeostasis and maintain a continuous Fe deficiency response in the Fe-efficient woody plant *M. xiaojinensis*. More broadly, this study reveals a new mechanism in which ROS mediate both positive and negative regulation of plant responses to Fe deficiency stress.

## Introduction

Fe is an essential micronutrient required for a wide variety of cellular functions in plant growth and development. Although Fe is abundant in the soil, Fe availability is limited due to its poor solubility in an oxygen-rich atmosphere (Briat et al., 1995; Crichton and Pierre, 2001; Darbani et al., 2013). Plants have evolved two distinct strategies of Fe uptake to cope with Fe deficiency (Marschner et al., 1986; Römheld and Marschner, 1990; Briat et al., 1995). Non-graminaceous plants use a reduction strategy that involves the reduction of ferric chelates at the root surface and the absorption of the ferrous Fe across the root plasma membrane (Strategy I), whereas graminaceous plants secrete phytosiderophores to enhance Fe uptake from the soil (Strategy II) (Marschner, 2011). In plants employing Strategy I, the regulation of root Fe (III) reductase and proton extrusion activity are thought to be crucial events controlling Fe acquisition, and the roles of auxin, NO, and ethylene in the regulation of Fe deficiency responses are widely accepted (Schmidt and Bartels, 1996; Romera and Alcántara, 2004; Chen et al., 2010; Wu et al., 2012).

The main reactive oxygen species (ROS) include non-radical molecules such as singlet oxygen (^1^O_2_) and hydrogen peroxide (H_2_O_2_), as well as free radicals such as superoxide (O_2_^-^) and hydroxyl radicals (•OH) (da Silva et al., 2008). Besides their harmful effects, ROS act as signaling molecules that regulate plant development, and biotic and abiotic stress responses (Mittler et al., 2004). Recent research has focused on ROS metabolism (Krasensky and Jonak, 2012; Noctor et al., 2014), and sensory and signaling networks (Dietz, 2008; Miller et al., 2010; Krasensky and Jonak, 2012; Baxter et al., 2014), as well as the cross-talk with other signaling pathways (Suzuki et al., 2012; Noctor et al., 2014).

As Fe is one constituent of the electron transport chain in mitochondria and chloroplasts, Fe deficiency can result in an imbalance of cellular redox. The role of ROS in Fe response regulation has not been well-defined, and they may play multiple roles. Glutathione (GSH) and ascorbate (ASC) act as ROS scavengers, which protect *Arabidopsis* seedlings from Fe deficiency, preserving cellular redox homeostasis and improving internal Fe availability (Ramírez et al., 2013). In addition, GSH and ASC levels were increased in cucumber and sugar beet exposed to conditions of Fe deficiency (Zaharieva et al., 1999; Zaharieva and Abadìa, 2003). H_2_O_2_ is involved in the regulation of ferritins in response to excess Fe to alleviate oxidative stress in leaves (Ravet et al., 2009; Briat et al., 2010), flowers (Sudre et al., 2013), and roots (Ravet et al., 2012; Reyt et al., 2015). ROS production has also been demonstrated under Fe deficiency in sunflower and maize (Ranieri et al., 2001; Sun et al., 2007). ROS could also be linked with Fe deficiency regulation since they have been found associated with NO and ethylene in abiotic stress signaling (Brumbarova et al., 2015; Xia et al., 2015). Recently, an abiotic stress-induced transcription factor, ZAT12, was identified, which functions as a negative regulator of Fe acquisition, and the authors suggested H_2_O_2_ mediates the negative regulation of plant responses to prolonged stress (Le et al., 2016). Thus, the role of ROS in the regulation of Fe deficiency responses needs to be investigated further.

Some species, such as tomato (*Solanum lycopersicum*), lettuce (*Lactuca sativa*), peanut (*Arachis hypogaea*), and apple rootstock (*Malus xiaojinensis* and *Malus baccata*), show different levels of resistance to Fe deficiency among genotypes (Han et al., 1994, 1998; Wang and Li, 1995; Xu et al., 2009; Sun et al., 2013; Xia et al., 2014). Self-incompatible species, such as apple, have higher levels of genetic variation, and the use of highly heterozygous *Malus* genotypes allowed us to identify an Fe-efficient woody plant in which to study the role of ROS in the response to Fe deficiency. We proposed a model that Fe deficiency might trigger ROS production, which would then act as an early response signal to mediate and maintain an Fe deficiency-induced response.

## Results

### Fe Deficiency Induces ROS Production at an Early Stage and then Activates ROS Scavenging Mechanisms in *M. xiaojinensis*

**FIGURE 1:**
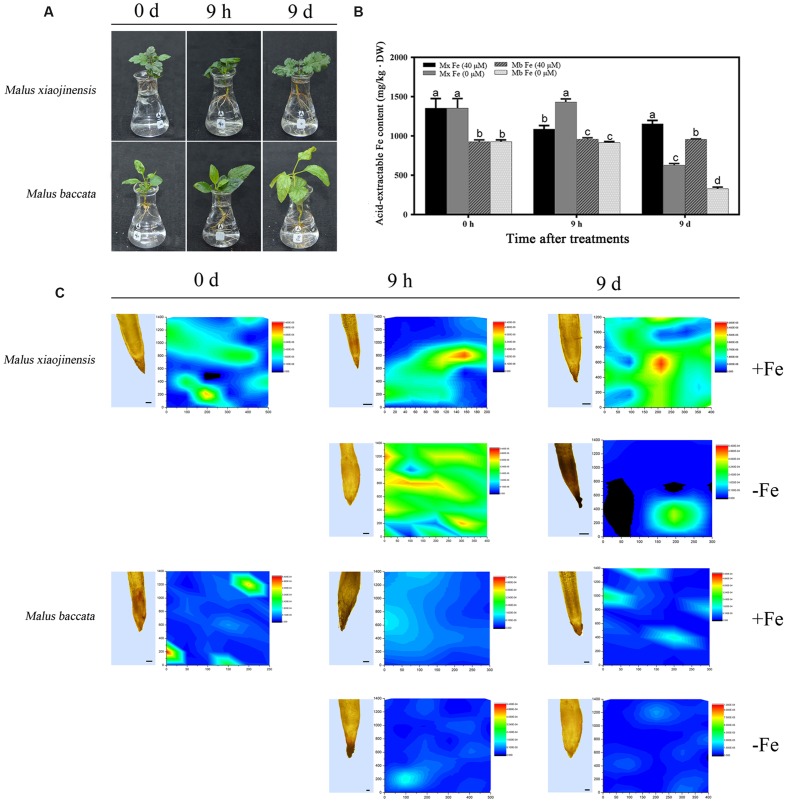
**Active Fe content and Fe distributions in roots, and leaf chlorosis of *Malus xiaojinens* and *Malus baccata* with Fe-sufficient (+Fe) and Fe-deficient (-Fe) treatment.** **(A)** Phenotype of *M. xiaojinensis* and *M. baccata* grown in Fe-deficient conditions for 0 days, 9 h, and 9 days. **(B)** Active Fe contents in roots. Vertical bars are mean ± SE (*n* = 3). Bars carrying different letters are significantly different (Duncan’s multiple-range test, *p* < 0.05). **(C)** Longitudinal sections of root top section samples observed by microscope (left; bars = 100 μm) and synchrotron radiation X-ray fluorescence (SR-mXRF) scanning images of the respective samples (right). The samples were treated with +Fe (40 mM FeNa-EDTA), or -Fe (0 Mm FeNa-EDTA) for 0 days, 9 h, and 9 days. The color of the bars from blue to red indicates the Fe content from low to high.

Both *M. xiaojinensis* and *M. baccata* are valued in China as native apple rootstocks. *M. xiaojinensis* performs Fe uptake with high efficiency (Han et al., 1994, 1998, 2005). However, compared with that in *M. xiaojinensis*, the Fe uptake efficiency in *M. baccata* is much lower. As shown in **Figure 1**, typical Fe deficiency symptoms resulting from low Fe treatment for 9 days were quite obvious in *M. baccata* but not in *M. xiaojinensis* (**Figure 1A**). *M. xiaojinensis* had higher active Fe content in roots than did *M. baccata*, indicating a variation in Fe uptake ability between the two *Malus* genotypes (**Figure 1B**). Further, our microtomography analysis of Fe distribution in roots of the two species confirmed this difference. The X-ray fluorescence (XRF) maps of the Fe distribution pattern in the roots showed the Fe content in *M. xiaojinensis* roots was higher than that in *M. baccata* roots (**Figure 1C**).

**FIGURE 2:**
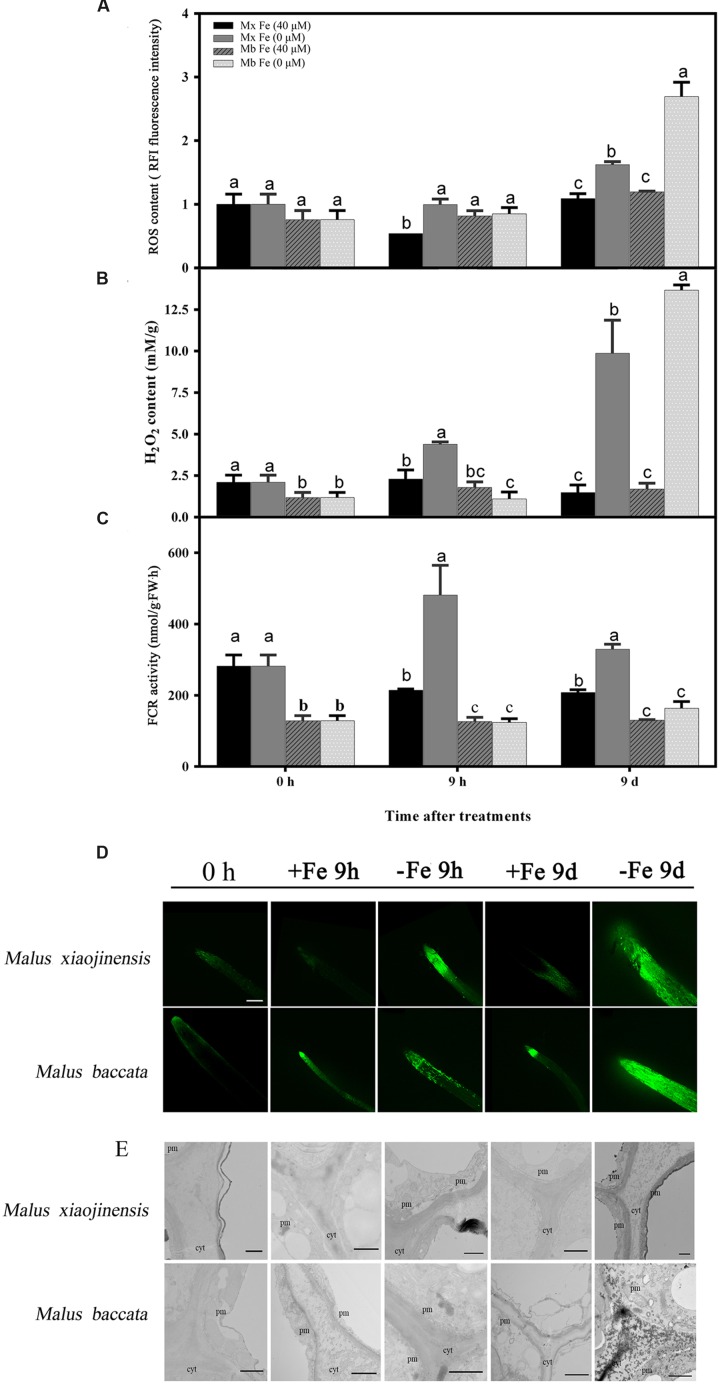
**Reactive oxygen species (ROS), H_2_O_2_ content, ferric-chelate reductase (FCR) activity, and tissue localization in the roots of *M. xiaojinensis* (Mx) and *M. baccata* (Mb) plants with Fe-sufficient (+Fe) and Fe-deficient (-Fe) treatment.** **(A)** ROS content in root tips (3–5 mm). **(B)** H_2_O_2_ content in roots. **(C)** FCR activity in roots. Values represent the mean and standard error of three replications. The dashed line is the critical value. **(D)** ROS system localization shown as green fluorescence from DCFH-DA in roots, bar = 500 μm. **(E)** Cytochemical localization of root tip; cyt, cytoderm; cm, cytomembrane. Bar = 1 μm. Vertical bars are mean ± SE (*n* = 3). Bars carrying different letters are significantly different (Duncan’s multiple-range test, *p* < 0.05).

The ROS production in roots determined by DCFH-DA fluorescence was intensified at an early stage of Fe deficiency and then weakened after prolonged Fe deficiency (**Figure 2D**). H_2_O_2_ localization in the root was monitored by reaction of CeCl_3_. A clear signal was observed in the apoplast, particularly in the root of *M. baccata* at the prolonged Fe deficiency stage (**Figure 2E**).

**FIGURE 3:**
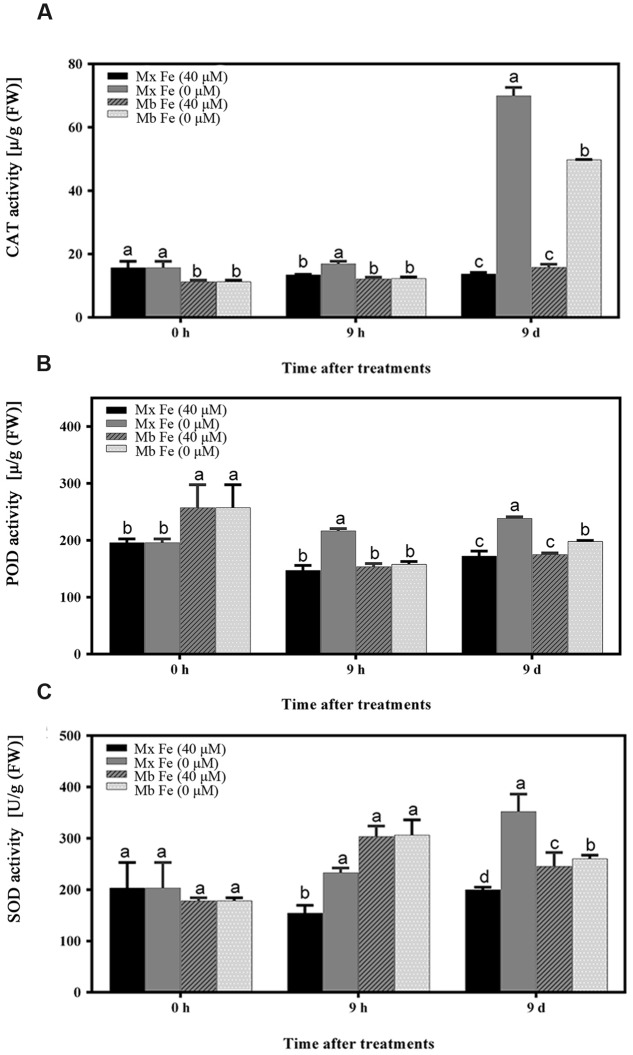
**Oxidative stress-related enzyme activities in root tissues.** **(A)** CAT enzyme activity of root tissues. **(B)** POD enzyme activity of root tissues. **(C)** SOD enzyme activity of root tissues. The values represent the mean and standard error of three replications. Vertical bars are mean ± SE (*n* = 3). Bars carrying different letters are significantly different (Duncan’s multiple-range test, *p* < 0.05).

The hypothesis that Fe deficiency can trigger ROS production was then tested. Total ROS and H_2_O_2_ were quantified in roots of *M. xiaojinensis* and *M. baccata* exposed to Fe deficiency. As shown in **Figures 2A,B** and **Supplementary Figure S1**, Fe deficiency was capable of triggering ROS and H_2_O_2_ production at the early Fe-deficient stage (9 h) in *M. xiaojinensis*, but not in *M. baccata*. Interestingly, after prolonged exposure to Fe deficiency, the ROS and H_2_O_2_ content was reduced in *M. xiaojinensis* after 1–3 days and was not significantly different from that in the Fe-sufficient treatment. This result was not observed in *M. baccata*. Catalase (CAT), surperoxide dismutase (SOD), and peroxidase (POD) can act as ROS scavengers, keeping cellular redox homeostasis under control. Total CAT, POD, and SOD levels quantified in roots exposed to Fe deficiency were increased at 9 h in *M. xiaojinensis* but were not affected in roots of *M. baccata* (**Figure 3**). These results suggest Fe deficiency can trigger ROS scavengers in order to maintain the cellular redox homeostasis in the early stage of Fe deficiency.

These results demonstrate that Fe deficiency is indeed capable of causing a significant accumulation of ROS in roots; however, the Fe-efficient species *M. xiaojinensis* could activate scavenging mechanisms to preserve the redox homeostasis during prolonged Fe deficiency treatment.

### Up-Regulation of the Fe Deficiency-Induced Response is Associated with Systemic ROS Production at an Early Stage

**FIGURE 4:**
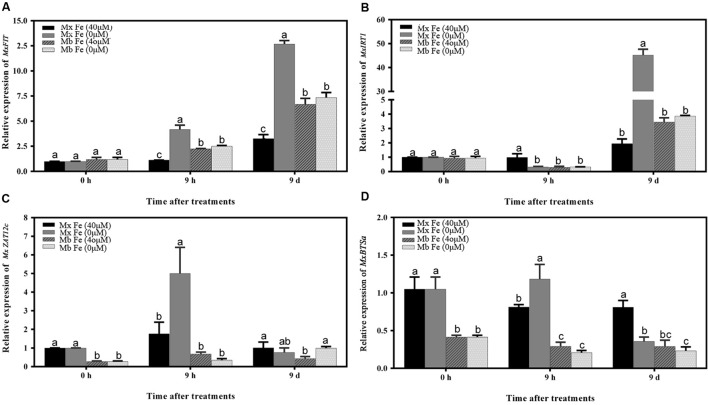
**Time course of the expression of different genes under Fe sufficiency and deficiency conditions in *M. xiaojinensis* and *M. baccata* roots.** The vertical axis represents the relative gene expression ratios; the horizontal axis represents the times after treatment. The values represent the mean and standard error of three replications. Vertical bars are mean ± SE (*n* = 3). Bars carrying different letters are significantly different (Duncan’s multiple-range test, *p* < 0.05). **(A)** The relative expression of *MxFIT* gene. **(B)** The relative expression of *MxIRT1* gene. **(C)** The relative expression of *MxZat12c* gene. **(D)** The relative expression of *MxBTSa* gene.

As shown in **Figure 2**, Fe deficiency induced a significant increase in root ROS contents. Consistent with ROS production, the results showed that Fe deprivation caused a significant increase in root Fe (III) reductase activity of *M. xiaojinensis* at 9 h (**Figure 2C**). An attempt was therefore made to assess whether the Fe deficiency-induced alterations in root Fe (III) reductase activity and proton extrusion were mediated by ROS signaling. It was found that the Fe deficiency-induced increase in root ROS levels was correlated with the increased expression of *MxFIT* and *MxBTSa* (**Figures 4A,D**). The *Arabidopsis* ortholog of the FER gene, FIT, was identified from the 161 predicted bHLH proteins by microarray analysis (Bauer et al., 2007). FIT also plays an important role in positively regulating various iron deficiency inducible genes, including IRT1 and FRO2 (Yuan et al., 2005; Bauer et al., 2007). BRUTUS (BTS), a putative E3 ligase protein, with metal ion binding and DNA binding domains, which negatively regulates the response to iron deficiency (Long et al., 2010).

**FIGURE 5:**
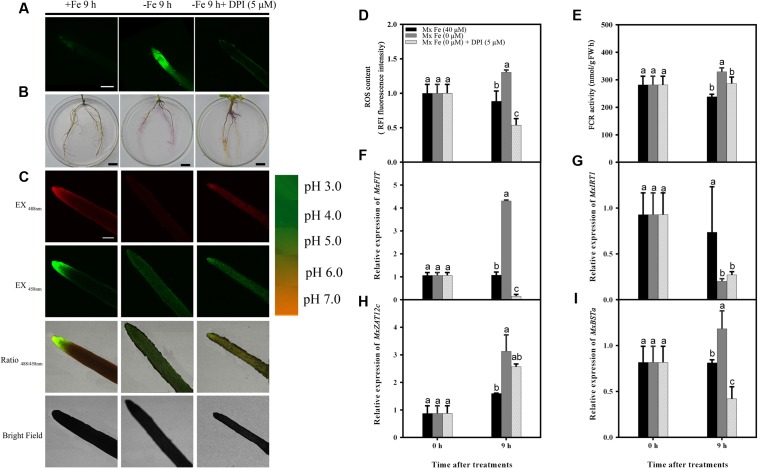
**Detection of ROS content, FCR activity, and the expression of different genes in *M. xiaojinensis* induced by DPI.** Seedlings were grown on Fe-sufficient (+Fe) or Fe-deficient (-Fe) medium, or -Fe medium with 5 μM DPI for 0 days and 9 h. **(A)** ROS system localization shown as green fluorescence from DCFH-DA in roots, bar = 500 μm. **(B)** Visualization of FCR activity in roots (bars = 1 cm). **(C)** Emission intensities of roots loaded with the pH-sensitive fluorescent dye BCECF at 488 nm (top, red) and 458 nm (center, green). The ratio images show a red fluorescence indicating increased root pH in -Fe medium with 5 μM DPI compared with -Fe medium. The pseudo-color scale on the right indicates the intensity of fluorescence in which yellow and red represent minimum and maximum intensity, respectively. Bar = 500 μm. **(D)** ROS content of root tips (3–5 mm). **(E)** FCR activity of roots. **(F–I)** The expression of different genes in *M. xiaojinensis* roots under early stage iron stress. Vertical bars are mean ± SE (*n* = 3). Bars carrying different letters are significantly different (Duncan’s multiple-range test, *p* < 0.05).

Next, it was determined whether ROS inhibitors blocked the Fe deficiency responses. The responses to Fe deficiency were markedly repressed by exposure to diphenyleneiodonium (DPI), which can block ROS production (**Figures 5A–C**). DPI application strongly inhibited the Fe deficiency-induced increase in reductase activity (**Figures 5B,E**) and proton extrusion (**Figure 5C**). These results imply that a certain endogenous ROS level is necessary for the Fe deficiency-based induction of expression of transcription factors, such as FIT (**Figure 5F**). Taken together, these results demonstrate that ROS play a critical role in Fe deficiency-induced signaling at an early stage.

#### *M. xiaojinensis* could Activate ROS Scavenging Mechanisms to Maintain a Persistent Fe Deficiency-Induced Response with Prolonged Fe Deficiency Treatment

**FIGURE 6:**
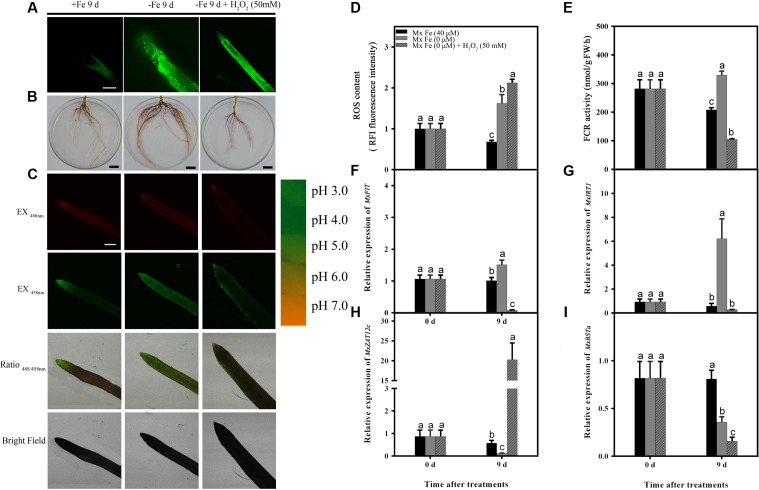
**Detection of ROS content, FCR activity, and the expression of different genes in *M. xiaojinensis* induced by H_2_O_2_.** Seedlings were grown on Fe-sufficient (+Fe) or Fe-deficient (-Fe) medium, or -Fe medium with 500 mM H_2_O_2_ for 0 and 9 days. **(A)** ROS system localization shown as green fluorescence from DCFH-DA in roots, bar = 500 μm. **(B)** Visualization of FCR activity in roots (bars = 1 cm). **(C)** Emission intensities of roots loaded with the pH-sensitive fluorescent dye BCECF at 488 nm (top, red) and 458 nm (center, green). The ratio images show a red fluorescence indicating increased root pH in -Fe medium with 50 mM H_2_O compared with -Fe medium. The pseudo-color scale on the right indicates the intensity of fluorescence in which yellow and red represent minimum and maximum intensity, respectively. Bar = 500 μm. **(D)** ROS content of root tips (3–5 mm). **(E)** FCR activity of roots. **(F–I)** The expression of different genes in *M. xiaojinensis* roots under the later stage of iron stress. Vertical bars are mean ± SE (*n* = 3). Bars carrying different letters are significantly different (Duncan’s multiple-range test, *p* < 0.05).

As described above, *M. xiaojinensis* and *M. baccata* are related plants but with a rather different ROS accumulation following prolonged Fe deficiency treatment. After 1 day of Fe deficiency, while the ROS content was decreased in *M. xiaojinensis* compared with the ROS content at 9 days (**Supplementary Figure S1**), both root Fe (III) reductase activity (**Figure 6B**) and proton extrusion (**Figure 6C**) were maintained at a higher level. Total CAT, POD, and SOD levels quantified in roots exposed to Fe deficiency were increased at day 9 in *M. xiaojinensis* and they were higher than those in roots of *M. baccata* during Fe deficiency (**Figures 3A–C**). These results suggest ROS scavengers are involved in maintaining the cellular redox homeostasis during prolonged Fe deficiency treatment.

Since H_2_O_2_ mediates the negative regulation of plant responses to prolonged Fe deficiency stress, H_2_O_2_ application experiments were performed in order to reveal the roles of ROS in the response of *M. xiaojinensis* to prolonged Fe deficiency stress. As shown in **Figure 6**, H_2_O_2_ application strongly inhibited the Fe deficiency-induced increase in reductase activity and proton extrusion at day 9. It was next determined whether the negative regulator *MxZAT12c* responded to H_2_O_2_ during prolonged Fe deficiency treatment; *MxZAT12c* was induced as expected (**Figure 6H**). Furthermore, H_2_O_2_ treatment prevented the response of *MxFIT*, *MxIRT1*, and *MxBSTa* to Fe deficiency (**Figures 6F,G,I**). From the expression pattern of these genes under H_2_O_2_ treatment, we deduced that ROS may act as a repressor signal of the Fe deficiency response upon prolonged Fe deficiency at day 9.

## Discussion

We hypothesize that Fe deficiency might trigger ROS production in the Fe-efficient woody plant *M. xiaojinensis*, which would then act as an early response signal to mediate and maintain an Fe deficiency-induced response. Some studies support the involvement of H_2_O_2_ in the regulation of ferritins in response to excess Fe to prevent oxidative stress (Ravet et al., 2009, 2012; Briat et al., 2010; Sudre et al., 2013; Reyt et al., 2015), but how ROS participate in that regulation of Fe deficiency stress in woody plants remained unknown. Previous research has proposed that H_2_O_2_ can be considered an intermediate in the auto-regulatory suppression of FIT after prolonged Fe deficiency in *Arabidopsis thaliana* (Le et al., 2016). In the present study, Fe deficiency responses were markedly repressed by exposure to a ROS inhibitor at an early stage, which suggests that H_2_O_2_ is an enhancer of the early Fe deficiency response in *M. xiaojinensis* (**Figure 5**).

Compared with *A. thaliana*, a relatively high level of variation has been maintained between, or among, domesticated crops and their close wild relatives (Huang and Han, 2014). Self-incompatible species, such as apple, have higher levels of variation, and the use of highly heterozygous *Malus* genotypes allowed us to demonstrate that ROS could be an early stage signal to enhance the efficiency of Fe uptake in Fe-efficient woody plants. By taking advantage of the distinct capabilities of two related woody species (*M. xiaojinensis* and *M. baccata*) to cope with Fe deficiency, we showed that ROS could be the hub for both positive and negative regulators dependent on an auto-regulatory loop requiring the ROS itself.

Our results showed that ROS content increased at an early stage of Fe deficiency treatment, then decreased with prolonged Fe starvation in order to maintain the cellular redox homeostasis. When the high levels of ROS generated exceed the possibility of being controlled by the cell’s antioxidant system, oxidative damage is caused (Allen, 1995). GSH and ASC levels have been shown to increase with the progression of Fe deficiency in sugar beet roots (Zaharieva et al., 2004). In *A. thaliana*, GSH and ASC supplementation preserved cellular redox homeostasis to protect *Arabidopsis* seedlings from Fe deficiency (Ramírez et al., 2013). Our results suggest that the roots trigger a primary initiation of ROS signaling under Fe deficiency to enhance Fe uptake (**Figure 5**). However, during prolonged Fe deficiency, the identification of ROS scavengers along with decreased effects of ROS provided a clue as to how this initial ROS production is counterbalanced at the molecular level, possibly to avoid oxidative damage in Fe-efficient woody plants (**Figures 4** and **6**).

**FIGURE 7:**
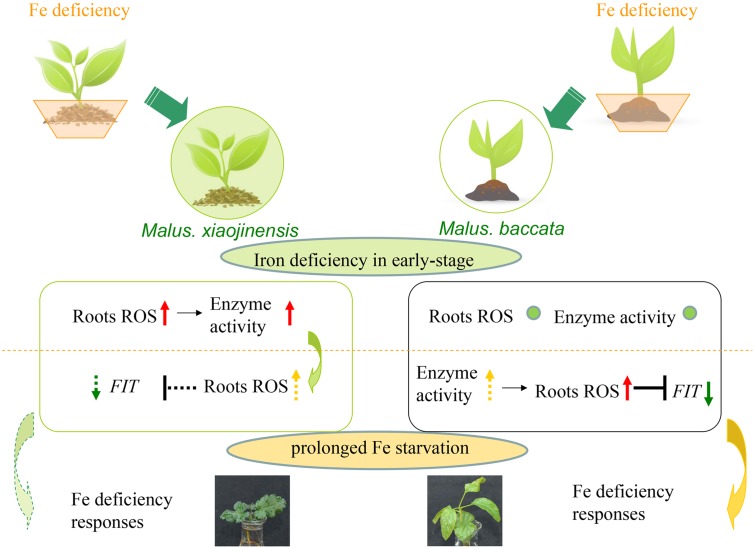
**Model of signaling in response to Fe deficiency in Fe-efficient and Fe-inefficient woody plants.** *M. xiaojinensis* is an Fe-efficient woody plant represented by a filled green circle; *M. baccata* is an Fe-inefficient plant represented by an unfilled circle. The lower portion of the diagram shows the signaling changes in the roots of the two plants. The red up-arrow indicates a significant increase; the yellow dotted line up-arrow indicates slow growth; no obvious change is represented by a small green circle; the green down-arrow indicates a significant reduction; the green dotted line down-arrow indicates a gradual reduction; the black ‘T’ indicates inhibition or repression; the dotted line ‘T’ indicates a delayed effect of inhibition. The orange dotted line represents the boundary of iron deficiency in the early stage and prolonged Fe starvation.

As Fe is a constituent of enzymes associated with the cellular antioxidant system such as ascorbate peroxidase (APX), CAT, POD, and Fe SOD, plants exposed to Fe deficiency would be more sensitive to oxidative stress (Kumar et al., 2010). In mulberry, maize, and cauliflower it has been suggested that Fe deficiency causes a decrease in CAT, POD, and APX activities (Tewari et al., 2006). Here, it was demonstrated CAT, POD, and SOD levels increased during prolonged Fe deficiency (**Figure 3**). We therefore deduced that in the Fe-efficient woody plant *M. xiaojinensis*, but not the Fe-inefficient woody plant *M. baccata*, ROS function mediated the primary Fe deficiency-induced physiological responses. With prolonged Fe deficiency treatment, plants need to cope with elevated ROS production (**Figure 7**). This underlies the need for a negative regulator of ROS production, such as the scavenging mechanism.

Reactive oxygen species production and associated redox processing are an integral part of hormone regulation and function in the control of plant development and stress tolerance. ROS could also be linked with Fe deficiency regulation since they have been found connected with NO and ethylene in abiotic stress signaling (Brumbarova et al., 2015; Xia et al., 2015). The stress-induced accumulation of ROS may alter auxin signaling through oxidative inactivation or degradation of auxin, and also be involved in auxin signaling and polar auxin transport (Blomster et al., 2011; Peer et al., 2013; Xia et al., 2015). Previous work in our laboratory showed the Fe deficiency-induced physiological responses are mediated by systemic auxin signaling (Wu et al., 2011). In the present study, we suggested that the ROS could be involved in early stage signaling of the Fe-deficiency responses. Although, the relationship between the auxin and ROS protective responses is not clear, ROS appear to promote stress tolerance by cooperating with auxin. However, as yet, no auxin-ROS that cooperate in response to environmental stresses have been identified.

Based on the data presented in this study, a model is proposed for the early stage signaling of the Fe deficiency responses in Fe-efficient woody plants (**Figure 7**). Our results suggest that the roots first function to perceive the Fe deficiency, which triggers a primary initiation of ROS signaling. The initial ROS production functions to mediate the corresponding responses in the roots only in the Fe-efficient *M. xiaojinensis* and not the Fe-inefficient *M. baccata*. Further, once the ROS production has been initiated, with prolonged Fe starvation ROS scavenging mechanisms are activated in *M. xiaojinensis* that are absent in *M. baccata*, which could represent a feedback repression loop for ROS to preserve redox homeostasis and maintain continuous Fe deficiency responses.

Our study reveals a new mechanism in which ROS mediate both the positive and negative regulation of plant responses to Fe deficiency stress (**Figure 7**). Undoubtedly, this work will trigger broad future research to elucidate the mechanisms of how plants adapt to the deficiency of important metal ions.

## Materials and Methods

### Plant Materials and Growth Conditions

*Malus xiaojinensis* seedlings were propagated on Murashige and Skoog medium (MS) (Huayueyang Biotechnology, Co, Ltd, Beijing, China) containing 0.5 mg/L 6-benzylaminopurine and 0.3 mg/L indole-3-butyric acid (IBA) for 1 month, and then transferred to ½ MS medium containing 0.5 mg/L IBA for 1 month for rooting. The rooted seedlings were transferred to Hoagland’s nutrient solution (Gao et al., 2011). The pH was adjusted to pH 6.0 with 1 M NaOH. The solution was refreshed every 6 days. Plants were grown in a growth room at 25 ± 2°C day/17 ± 2°C night with a 16 h photoperiod at a light intensity of 250 mmol quanta m^2^/s, 85% relative humidity. After 1 month, the plants were transferred into Fe-sufficient medium (40 μM FeNa-EDTA) or Fe-deficient medium (0 μM FeNa-EDTA) with 50 μM DPI or Fe-deficient medium (0 μM FeNa-EDTA) with 50 mM H_2_O_2_. The roots were collected at 0 days, 9 h, and 9 days during these treatments. A completely randomized experimental design with three biological replicates and three plants for each replicate was used.

### Isolation of Total RNA and Synthesis of cDNA

Total RNA was isolated from the roots using an improved cetyltrimethylammonium bromide (CTAB) method (Gasic et al., 2004). The RNA was digested by DNase I (Takara Biotechnology, Co, Ltd, Dalian, China) and reverse transcribed by using an oligo-dT primer and reverse transcriptase (Takara Biotechnology, Co, Ltd, Dalian, China).

### ROS Localization and Quantification

Reactive oxygen species were imaged using DCFH-DA and a confocal laser scanning microscope (TE2000-E, Nikon, Co., Tokyo, Japan). Roots (3–5 mm from root tip) were loaded with 20 μM DCFH-DA in 20 mM HEPES/NaOH pH 7.5 buffer for 90 min, washed three times with fresh buffer, and analyzed microscopically (excitation 488 nm, emission 525 nm). The images were analyzed using EZ-C1 3.00 Free View software. For fluorescence quantification per gram of root tissue, roots were loaded with DCFH-DA and washed; the complete root system (approximately 50 mg) was subsequently ground in 0.5 mL HEPES/NaOH pH 7.5 buffer. The supernatants were then centrifuged (13, 000 × *g*, 10 min) at 4°C, and DCFH-DA concentration was determined by using a fluorescent spectrophotometer (F-7000, Hitachi, Tokyo, Japan) using excitation at 488 nm and emission at 525 nm. DCFH-DA was similarly processed without roots for comparison as a blank. Data are expressed as the mean ± SD of two independent experiments performed in triplicate.

### Quantification and *In situ* Localization of H_2_O_2_

Hydrogen peroxide was detected using the method described by Mukherjee and Choudhuri (1985). A root sample of 200 mg was ground with 1.5 mL pre-cooled acetone, then centrifuged (800 × *g*) at 4°C for 10 min. Then, 5% titanium sulfate and ammonia water were added to the supernatant, and it was centrifuged again (800 × *g*) at 4°C for 10 min. The precipitate was rinsed three times with acetone and vortexed. It was then redissolved in 10 M H_2_SO_4_. The absorbance of the supernatant at 415 nm was measured using an UV spectrophotometer (UV 1800, Shimadzu, Tokyo, Japan); 1.5 mL acetone was similarly processed without roots for comparison as a blank. The H_2_O_2_ content was determined based on a standard curve plotted using known H_2_O_2_ concentrations. The H_2_O_2_ content was measured as micromoles per gram fresh weight (μmol/g FW).

Freshly harvested roots were cut in 5 mm sections close to the root tip and subjected to protocols for cytochemical localization of H_2_O_2_. H_2_O_2_ production was assessed cytochemically via determination of cerium perhydroxide formation after reaction of CeCl_3_ with endogenous H_2_O_2_ (Bestwick et al., 1997). Root sections of *M. xiaojinensis* and *M. baccata* were incubated for 1 h in 5 mM CeCl_3_ in 50 mM 3-(*N*-morpholino) propanesulphonic acid (MOPS) pH 7.2, fixed in 1.25% glutaraldehyde and 1.25% paraformaldehyde in 50 mM sodium cacodylate buffer (CAB) pH 7.2 by vacuum evacuation of samples for 30 min, and after drying washed three times in CAB buffer for 10 min (Bestwick et al., 1997). Samples were then embedded in resin and observed by transmission electron microscopy (JEM-1230, JEOL, Tokyo, Japan).

### Enzyme Activity Assay

Root tissue (0.1 g) was frozen with liquid N_2_ and homogenized in 2.0 mL chilled 50 mM phosphate buffer (pH 7.0). The homogenate was centrifuged at 1, 3000 × *g*, for 20 min at 4°C. The supernatant was stored at 4°C and used for enzyme assays within 4 h.

Catalase activity was measured in a reaction mixture (3.0 mL) containing 200 μL of the extract, 0.3 mL 0.1 mM H_2_O_2_, and 1 mL distilled water in 0.2 M phosphate (pH 7.8) containing 1% (w/v) insoluble polyvinylpyrrolidone (PVP). The decrease in absorbance at 240 nm was monitored for 3 min, and the amount of enzyme digesting 1 μM substrate in 1 min was indicated as enzyme activity (μ) (Tewari et al., 2012). CAT activity was presented as μmol H_2_O_2_ decomposed/min (unit)/mg protein.

Peroxidase activity was estimated in a reaction mixture (3.0 mL) containing 50 μL 50 mM phosphate buffer (pH 7.0), 2.9 mL 0.3% guaiacol (v/v), 60 μL 0.004% H_2_O_2_ (v/v), and 50 μL root extract, and the change in absorbance was measured at 470 nm (Ranieri et al., 1997). Enzyme activity was expressed as ΔAbs/0.01 min (unit)/mg protein.

Superoxide dismutase activity was assayed by measuring its ability to inhibit the photochemical reduction of nitro blue tetrazolium (NBT) at 560 nm. The reaction mixture (3.0 mL) contained 1.5 mL 50 mM phosphate buffer (pH 7.8), 300 μL 750 μM NBT, 300 μL 20 μM riboflavin, 50 μL root enzyme extract, 300 μL 130 mM methionine (Met), and 1 mL distilled water (Bradford, 1976). The reaction mixture was exposed to light at 4000 Lx for 10 min. The amount of SOD corresponding to 50% inhibition of the reaction was defined as one unit of enzyme. SOD activity was expressed as U (unit)/mg protein.

### FCR Activity Determination and Visualization

The root FCR activity was quantified using a ferrozine assay (Schikora and Schmidt, 2001). All reduction assays were performed in 15 mL lightproof centrifuge tubes. The roots were initially washed in 0.5 mM CaSO_4_ for 5 min, and then the color reaction was carried out in a solution of 0.5 mM ferrozine, 0.5 mM FeNa-EDTA, and 0.5 mM CaSO_4_ at 25°C for 40 min. The reaction was adjusted to pH 6.0 with 1 M NaOH. Reduction activity was measured at 562 nm using a spectrophotometer (UV 1800, Shimadzu, Japan). The reduction rate was calculated after subtraction of the appropriate blanks (assay solution without roots). The rate of root FCR activity was calculated as moles of Fe^2+^-ferrozine per gram of fresh weight per hour. Data, expressed as the mean ± SD, represent three biological replicates, with each replicate containing four plant root samples. To visualize FCR activity spatially, the plant roots were initially pre-processed and then embedded in agarose (0.7%, w/v) medium containing the assay solution. Photographs were taken after 40 min (Li et al., 2000).

### Measurement of Root pH

Root pH of apple *M. xiaojinensis* was monitored with the cell-permeant and pH-sensitive fluorescent dye BCECF-AM (Tang et al., 2012). Root pH was quantified by a ratio analysis of the fluorescence at pH-dependent (488 nm) and pH-independent (458 nm) excitation wavelengths from a calibration curve (**Supplementary Figure S2**), and ratio images were produced using the ion concentration tool of Zeiss LSM confocal software.

### Measurement of Extra Iron Content

Approximately, 1 g of roots was maintained at 70°C for 7 days. One hundred milligrams of each dried sample was then treated with 10 ml 1.0 M HCl in an orbital shaker for 6 h, and the filtered extract was analyzed by polarized Zeeman atomic absorption spectrophotometry (Z-5000; Hitachi, Tokyo, Japan) (Takkar and Kaur, 1984; Han et al., 1994).

### Chemicals

DCFH-DA (Dichlorofluorescein diacetate) and BCECF-AM (3′-*O*-Acetyl-2′,7′-bis(carboxyethyl)-4) were purchased from Beyotime Institute of Biotechnology, Product codes S0033 and S1006^1^; DPI, HEPES, and ferrozine were from Sigma^2^.

#### Freeze-Drying Microtomy and SR-mXRF

Root samples of 1 cm length were cut off from the root tip and embedded quickly. The embedded root samples were sliced into 200 mm thick longitudinal and latitudinal sections with a freezing microtome (LEICA-CM 3050 S, Germany) and placed on Kapton tape, then freeze-dried (LGJ-10B, Beijing Four-Ring Science Instrument Factory) for about 24 h for SR-mXRF analysis.

The SR-mXRF microspectroscopy experiment was performed at 4W1B end station, Beijing synchrotron Radiation Facility, which runs a 2.5 GeV electron beam with a current from 150 to 250 mA. The incident X-ray energy was monochromatized by a W/B4C Double-Multilayer Monochromator at 15 keV and was focused down to 50 μm in diameter by the polycapillary lens. The two-dimensional mapping was acquired by step mode: the sample was kept on a precision motor-driven stage, scanning 50 μm stepwise for latitudinal samples and 100 μm stepwise for longitudinal samples. The Si (Li) solid-state detector was used to detect X-ray fluorescence emission lines with live time of 60 s. The data reduction and process were performed using the PyMCA^3^ package (Solé et al., 2007).

### Analysis of Gene Expression

The quantitative measurement of gene expression was performed using the cDNA samples with an AB7500 Real-time PCR System and the SYBR Green fluorescence dye (Takara) (Li et al., 2012).

The genes analyzed were the following: Fe deficiency-induced transcription factor *MxFIT1* (NCBI: XP_008360009.1), Fe^2+^ transporter *MxIRT1* (NCBI: AAO17059.1), *Malus* × *domestica* zinc finger protein *MxZAT12c* (NCBI: XM_008371622), putative E3 ligase BRUTUS *MxBTSa* (NCBI: XP_008393323.1). The sequences of all of the genes in the apple genome were obtained from the website http://genomics.research.iasma.it/ via its BLAST (basic local alignment search tool) service. Based on the BLASTP results in NCBI^4^ for those proteins encoded by the genes examined, primers were designed using Primer Premier 5 (Primer, Co., Canada). The β*-Actin* was used as the reference gene. The primer sets used are listed in Supplementary Table S1.

### Statistical Analysis

Statistical analysis was performed using the Statistical Product and Service Solutions (SPSS) software (IBM, Co., Armonk, NY, USA). All experimental data were tested by analysis of variance (ANOVA) and Duncan’s multiple-range test.

## Author Contributions

YW and ZH conceived and designed research. CS, TW, and LZ conducted experiments. DL, XZ, XX, and HM contributed new reagents and analytical tools. CS and TW wrote the manuscript. All authors read and approved the manuscript.

## Conflict of Interest Statement

The authors declare that the research was conducted in the absence of any commercial or financial relationships that could be construed as a potential conflict of interest.

## Footnotes

1 http://www.beyotime.com/goods.do?method=getmain

2 http://www.sigmaaldrich.com/

3 http://sourceforge.net/projects/pymca/

4 http://www.ncbi.nlm.nih.gov/

## Supplementary Material

**FIGURE S1 | Reactive oxygen species and H_2_O_2_ content of *M. xiaojinensis* (Mx) and *M. baccata* (Mb) plants with Fe-sufficient (+Fe) and Fe-deficient (-Fe) treatment.** **(A)** ROS content in root tips (3–5 mm). **(B)** H_2_O_2_ content in roots.

**FIGURE S2 |*In situ* calibration of BCECF-AM in *M. xiaojinensis* root tips.** **(A)** *M. xiaojinensis* root tips loaded with BCECF were imaged at 488 nm (top row, red) and 458 nm (second row, green) excitation of BCECF. Ratio images were pseudo-colored to show the pH gradient (third row). Bright-field images were also captured from the same root samples (bottom row). Bar = 500 μm. **(B)** The fluorescence ratios (488/458 nm) were plotted against the pH of the equilibration buffers to generate a calibration curve.
