# Supplementary figures and images for: Reactive Oxygen Species Function to Mediate the Fe Deficiency Response in an Fe-Efficient Apple Genotype: An Early Response Mechanism for Enhancing Reactive Oxygen Production
Source: Front Plant Sci. 2016 Nov 16;7:1726. doi: 10.3389/fpls.2016.01726 (PMC5110569; doi:10.3389/fpls.2016.01726)

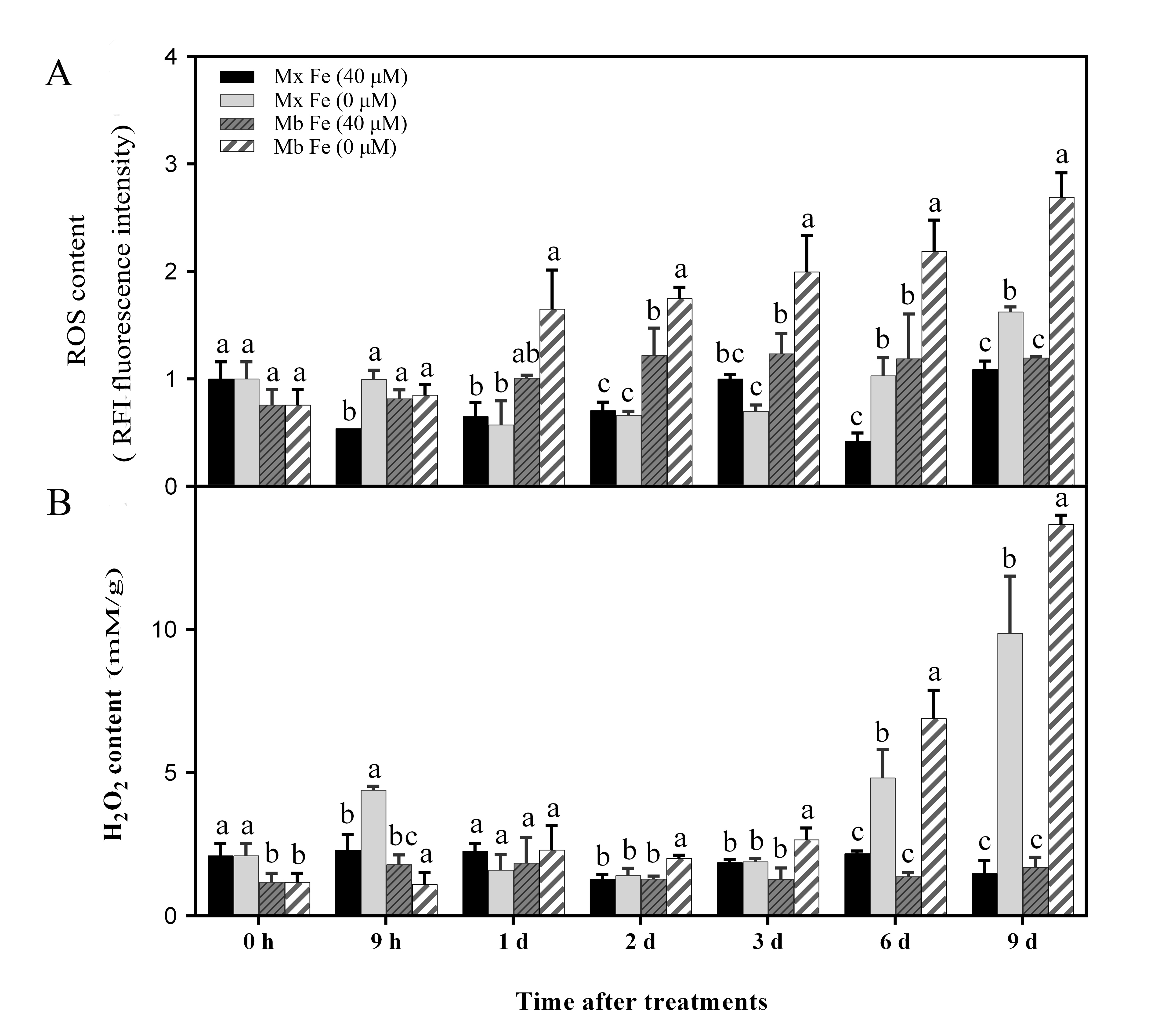

Supplement: Supplementary file 1 [file Image_1.jpeg]

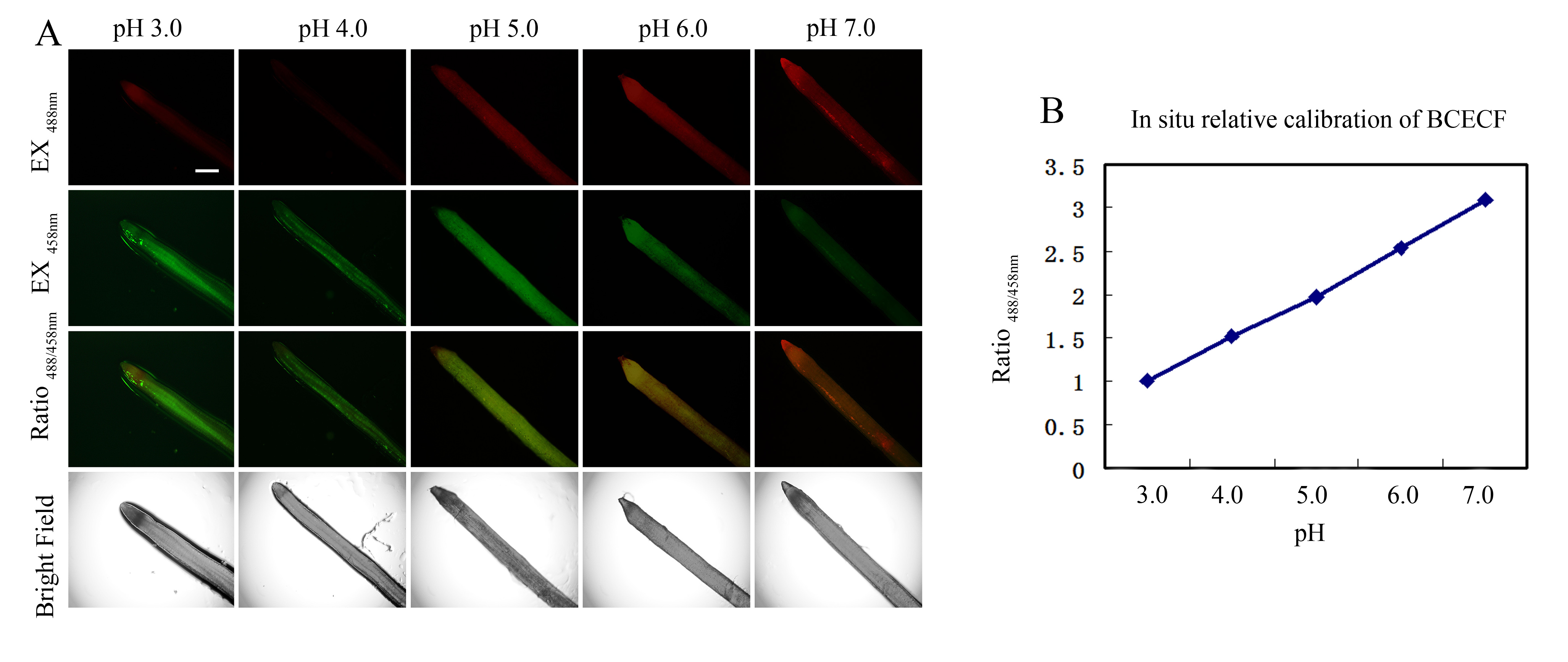

Supplement: Supplementary file 2 [file Image_2.jpeg]
