# Supplementary material for: Reactive Oxygen Species Function to Mediate the Fe Deficiency Response in an Fe-Efficient Apple Genotype: An Early Response Mechanism for Enhancing Reactive Oxygen Production
Source: Front Plant Sci. 2016 Nov 16;7:1726. doi: 10.3389/fpls.2016.01726 (PMC5110569; doi:10.3389/fpls.2016.01726)
Supplement: Supplementary file 3 [file Table_1.PDF]

Table 1 Primer sequences for the quantification of transcripts by real-time PCR.

Gene	Primer sequence
MxFIT	Fwd 5’-GGGAAACCATCAAGGAGGTCATA-3’
	Rev 5’-AGCCATTCATCATAAGGTCAGGA-3’
MxIRT1	Fwd 5’-TTGACAAGGGAGAAAACGGAGAC-3’
	Rev 5’-AACAACTGAATGGACAATGATACCC-3’
MxZAT12c	Fwd 5’-TCACAAAGCGAGCCACAA-3’
	Rev 5’-AACCGGCCATGTCCATATTAG-3’
MxBTSa	Fwd 5’-TTGGCATGCTTGATGCATTATT-3’
	Rev 5’-GTGAGGTACCCTTTCGATCAC-3’
β-Actin	Fwd 5’-TGGTGAGGCTCTATTCCAAC-3’
	Rev 5’-TGGCATATACTCTGGAGGCT-3’
